# Inertial Sensor Algorithm to Estimate Walk Distance

**DOI:** 10.3390/s22031077

**Published:** 2022-01-29

**Authors:** Vrutangkumar V. Shah, Carolin Curtze, Kristen Sowalsky, Ishu Arpan, Martina Mancini, Patricia Carlson-Kuhta, Mahmoud El-Gohary, Fay B. Horak, James McNames

**Affiliations:** 1 Department of Neurology, Oregon Health & Science University, 3181 SW Sam Jackson Park Road, Portland, OR 97239, USA; arpan@ohsu.edu (I.A.); mancinim@ohsu.edu (M.M.); carlsonp@ohsu.edu (P.C.-K.); horakf@ohsu.edu (F.B.H.); 2 Department of Biomechanics, University of Nebraska at Omaha, 6001 Dodge St., Omaha, NE 68182, USA; ccurtze@unomaha.edu; 3 APDM Wearable Technologie—A Clario Company, 2828 S Corbett Ave, Ste 135, Portland, OR 97201, USA; kristen.sowalsky@ert.com (K.S.); mahmoud.el-gohary@ert.com (M.E.-G.); james.mcnames@ert.com (J.M.); 4 Department of Electrical and Computer Engineering, Portland State University, 1825 SW Broadway, Portland, OR 97201, USA

**Keywords:** 6MWT, inertial sensors, neurological disorders, 400 m walk test, 6MWD

## Abstract

The “total distance walked” obtained during a standardized walking test is an integral component of physical fitness and health status tracking in a range of consumer and clinical applications. Wearable inertial sensors offer the advantages of providing accurate, objective, and reliable measures of gait while streamlining walk test administration. The aim of this study was to develop an inertial sensor-based algorithm to estimate the total distance walked using older subjects with impaired fasting glucose (Study I), and to test the generalizability of the proposed algorithm in patients with Multiple Sclerosis (Study II). All subjects wore two inertial sensors (Opals by Clario-APDM Wearable Technologies) on their feet. The walking distance algorithm was developed based on 108 older adults in Study I performing a 400 m walk test along a 20 m straight walkway. The validity of the algorithm was tested using a 6-minute walk test (6MWT) in two sub-studies of Study II with different lengths of a walkway, 15 m (Study II-A, *n* = 24) and 20 m (Study II-B, *n* = 22), respectively. The start and turn around points were marked with lines on the floor while smaller horizontal lines placed every 1 m served to calculate the manual distance walked (ground truth). The proposed algorithm calculates the forward distance traveled during each step as the change in the horizontal position from each foot-flat period to the subsequent foot-flat period. The total distance walked is then computed as the sum of walk distances for each stride, including turns. The proposed algorithm achieved an average absolute error rate of 1.92% with respect to a fixed 400 m distance for Study I. The same algorithm achieved an absolute error rate of 4.17% and 3.21% with respect to an averaged manual distance for 6MWT in Study II-A and Study II-B, respectively. These results demonstrate the potential of an inertial sensor-based algorithm to estimate a total distance walked with good accuracy with respect to the manual, clinical standard. Further work is needed to test the generalizability of the proposed algorithm with different administrators and populations, as well as larger diverse cohorts.

## 1. Introduction

Walking is one of the most common and important daily activities for functional independence. Walking abnormalities are prevalent in elderly people and people with neurological disorders, leading to an elevated risk of falls and reduced quality of life [1,2,3]. Clinically, walking tests are widely used as standard assessments to identify and track impaired walking ability. Walking tests are usually performed either over a fixed time or over a fixed distance. Among the many measures used to quantify walking abnormalities like gait speed [4], the total distance walked is one of the most common measures to assess functional independence [5].

The six-minute walk test (6MWT) assesses distance walked over 6 min and is the most established outcome measure of aerobic capacity in clinical trials [5,6,7]. The 6MWT is an objective tool that is traditionally used in clinics to assess functional capacity in chronic obstructive pulmonary disease and congestive heart failure [6,7,8]. In addition, the 6MWT has been commonly used as a functional test of aerobic capacity and endurance [7,8,9,10], to monitor disease state [11], and to investigate an effect of an intervention [12]. The primary measure of a 6MWT is the 6-minute walk distance (6MWD), the total distance covered in 6 mins. The 6MWD is reduced by several types of diseases, including obstructive lung disease, heart failure, arthritis, neuromuscular disease, frailty, multiple sclerosis, and neurological disorders [13,14].

Although the 6MWT is easy to perform, it has some practical limitations. To start with, it is typically administered by trained personnel in a clinical setting. The test requires a dedicated corridor in a clinic, of length between 30 m and 50 m, and no shorter than 15 m^5^. It does not consider the time it takes to turn, which could greatly influence the score, particularly with shorter laps and in neurological (like parkinsonism) or aging groups in which turning is specifically impaired. The test also requires an administrator to observe the test and note the distance, which, in turn, may lead to human error from a distance set-up mistake, error counting laps, or measuring the total distance improperly. Finally, patients need to visit the clinic where the test is performed, and hence the 6MWT is performed infrequently. 

Recently, the use of wearable inertial sensors has made it possible to quantify mobility in the clinic and during daily life [15,16,17,18,19,20,21]. Wearable inertial sensors may be used to easily and accurately measure the total walking distance during a prescribed task. There is also the potential for collecting the 6MWT remotely in everyday settings; this has the benefit of a participant not having to be in a clinic/lab for testing and allows a prescribed walking test to be administered more frequently. Additionally, wearable inertial sensors show a potential to provide continuous monitoring of multiple and perhaps more sensitive variables of walking, which enables trends to be identified, making it easier to distinguish when health is deteriorating [22]. Furthermore, wearable sensors provide an opportunity to scale up multi-center clinical trials without an additional burden on clinical sites. Although there are many advantages of wearable inertial sensors, the adaptation in clinical settings is still limited due to lack of regulatory, ethical, infrastructure, training and standardization in data collection and analysis, and security challenges. As a first step to improve the standard clinical 6MWT, we present an objective, and validated algorithm for total distance walked from wearable sensors on the feet.

Various studies have used wearable sensors placed at different body parts to estimate the total distance walked during a walking test [23,24,25,26,27,28,29,30,31,32,33,34,35]. However, all of the commercial/custom algorithms used in these studies either require a priori information (age, height, weight) or calibration data to calculate the total distance walked. Furthermore, only one research group validated their proposed algorithm to calculate the total distance walked on independent cohorts with different protocols [28]. To overcome these limitations, here we present an objective, and validated algorithm for total distance walked from feet sensors that does not depend on anthropometric information or calibration to calculate the total distance walked. The main contribution of this study is to show how these zero-velocity (foot-flat) periods can be used to estimate the horizontal distance traveled and to provide an assessment of how well this matches the straight-line distance typically used as the reference measure in clinical studies. Specifically, we tested the validity and generalizability of our proposed distance-walked algorithm in two independent cohorts (see Study II). Study I (*n* = 108) with a fixed distance walk test (400 m) in subjects over 65-years-old was used to develop the algorithm, while Study II with a fixed time walk test (6MWT) was used to validate and test the generalizability of the proposed algorithm in patients with multiple sclerosis. Study II had two sub-studies with different lengths of walkways, 15 m (Study II-A, *n* = 24) and 20 m (Study II-B, *n* = 22), respectively. 

## 2. Methods

### 2.1. Participants

Study I: 400 m fast walk with 20 m walkway (Algorithm Development Dataset). Older adults with impaired fasting glucose (IFG) were recruited on a convenience basis from the Portland VA Healthcare System (PORVAHCS). Inclusion criteria were: (a) ambulatory adults ≥65 years old with IFG, (b) sedentary, (c) weight-stable, (d) no walking aides, (e) no neurological conditions. Laboratory assessment was performed for fasting glucose to identify participants with IFG (100 mg/dL ≤ fasting glucose < 126 mg/dL). Exclusion criteria for Study I were medical conditions that are relative contraindications to metformin, increase the risk of major bleeding with muscle biopsies and affect muscle mass or performance measurements. The experimental protocol for Study I was approved by the Institutional Review Board of the PORVAHCS (#8860). All the participants provided informed written consent.

Impaired fasting glucose (>100 mg/dL) can be a precursor to diabetes mellitus (>126 mg/dL), which is associated with peripheral neuropathy, retinopathy, and peripheral artery disease that can impair gait at the time of diagnosis of diabetes. However, the population-based, Rotterdam study on 3019 adults showed that people with elevated fasting glucose had normal gait characteristics unlike those with diabetes, who had abnormal gait characteristics, so they can be considered an elderly control group [36].

Study II (A and B): A 6MWT with a 15 m Walkway (Algorithm Validation Dataset A) and a 20 m Walkway (Algorithm Validation Dataset B). As the objective of this study was to test the generalizability of the proposed algorithm, in addition to a fixed distance protocol (Study I: 400 m fast walk), we have included two sub studies (Study II: A and B) of a fixed time protocol (6MWT) with different walkway length.

People with Multiple Sclerosis (PwMS) and age-matched healthy controls (HC) were recruited on a convenience basis from the Oregon Health & Science University—MS Clinic and the local community. Inclusion and exclusion criteria were the same for both sub-studies of Study II (A and B). Inclusion criteria were ages 18–65 years, an absence of any orthopedic or neurologic problems other than Multiple Sclerosis (MS), and the ability to walk for 6 min without an assistive device. Exclusion criteria were MS exacerbation or the use of corticosteroids within 30 days of screening. Participants were instructed not to take caffeine in the morning of the testing and all testing was done between 10 am to noon. Additionally, PwMS were told not to take fatigue-related medication for 24 h before testing. The experimental protocol for Study II (A and B) was approved by the Institute Review Board of the Oregon Health & Science University (#15568 and #18714). All the participants provided informed written consent.

All the participants in both the studies (study I and II) gave written informed consent in accordance with the Declaration of Helsinki.

### 2.2. Data Collection

**Figure 1:**
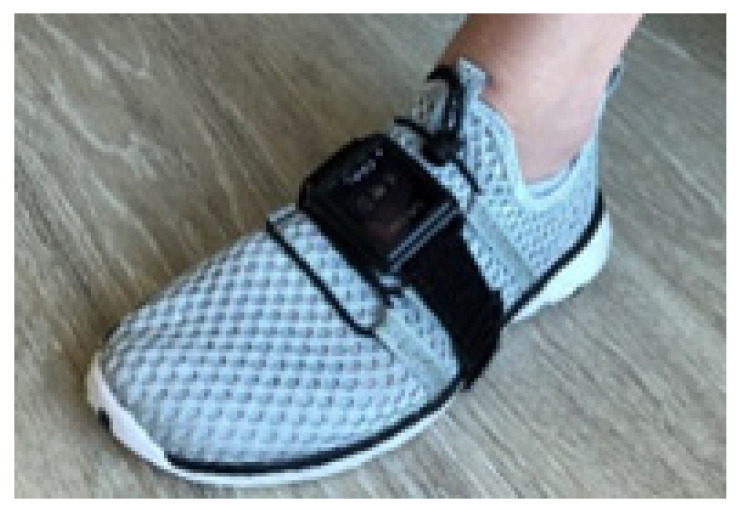
Inertial sensor (Opal) placement on the foot dorsum.

Inertial sensor placement for all studies. The subjects wore six inertial sensors (Opals by Clario—APDM Wearable Technologies, Portland, OR, USA) that included triaxial accelerometers, gyroscopes, and magnetometers. The sensor data were sampled at 128 Hz. The sensors were attached to the dorsum of both feet (Figure 1), wrists, sternum, and lumbar area. Only sensor data from the feet were used in this analysis.

Protocol for Study I (400 m walk test). Cones were placed on the floor 20 m apart and participants were instructed to walk 10 laps as fast as possible, making clockwise turns around the cones. The 400 m distance is considered a ground truth to compare the results with the total distance walked from the proposed algorithm.

Protocol for Study II (6MWT). Participants were instructed to complete the 6MWT at their fastest speed, aiming to cover as much distance as possible [37] by walking back-and-forth along a 15 m straight walkway (for Study II-A) and along a 20 m straight walkway (for Study II-B). The walkway had a start line, placed horizontally on the floor at the beginning, with smaller horizontal lines placed every 1 m to calculate a total distance walk. The manually calculated total distance walked using a tape measure is considered a ground truth to compare the results with the total distance walked from the proposed algorithm.

### 2.3. Total Distance Walked Algorithm

**Figure 2:**
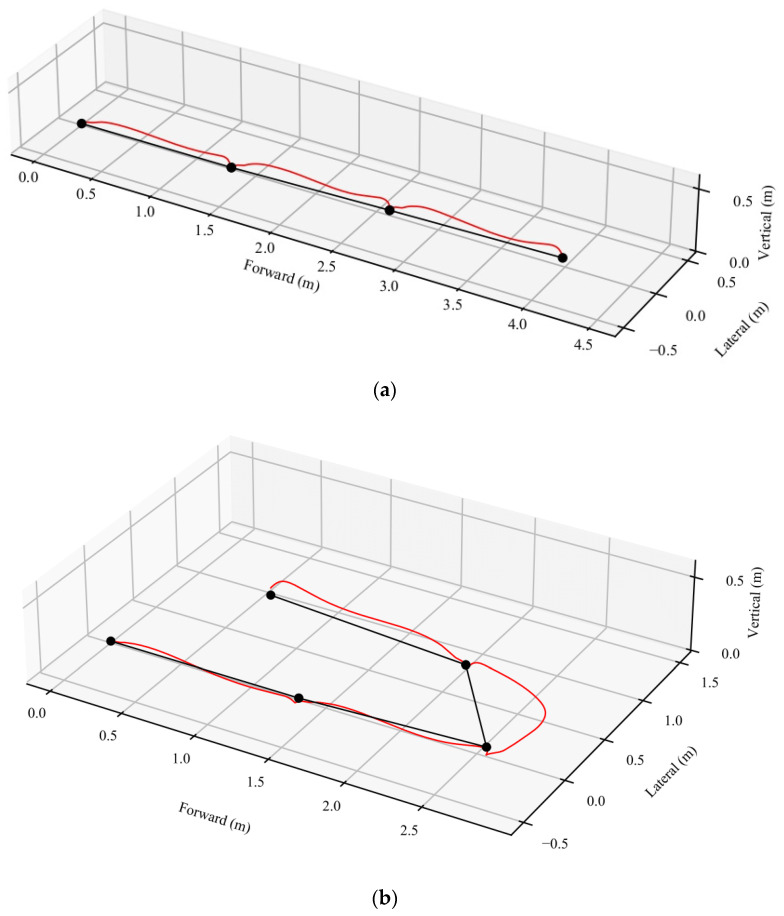
Example of walk trajectory used to calculate the total distance walked on a straight path (**a**) and with addition of a turn (**b**). The black dots show the position of the foot during the foot flat periods, the red trace shows the three-dimensional trajectory of the foot during each stride, and the black line segments show the horizontal distance traveled during each of the three strides. The total walk distance is then simply computed as the sum of walk distances for each stride.

We used APDM’s Mobility Lab algorithms that have been validated previously [38,39] to estimate the entire, three-dimensional trajectory of the foot, including its orientation in space. This is determined by first estimating the orientation of the sensors by fusing the rotational rate estimated by the gyroscopes with the gravitational component exhibited in the accelerometers using well-known methods [40,41,42]. This orientation can be used to express the accelerometer signals in an Earth reference frame so that the acceleration due to gravity can simply be subtracted. Estimates of the velocity and position can then be obtained directly by numerical integration, though any error in the acceleration estimates accumulates rapidly during this process. To help reduce this effect, the algorithm detects periods when the foot is in contact with the ground and the velocity of the sensors is known to be zero to update the velocity estimates. This approach is well known for using zero-velocity updates [40,42]. Once the spatial-temporal trajectories are known, we implemented further calculations to determine the horizontal distance traveled [43,44]. We calculated the forward distance traveled during each step as the change in the horizontal position from each foot-flat period to the subsequent foot-flat period. Specifically, let $v\left(t\right)={\left[x\left(t\right),\;y\left(t\right),\;z\left(t\right)\right]}^{T}$ be a three-dimensional vector with the spatial coordinates in an Earth reference frame at each time t. In this coordinate frame, the last element of the vector represents the vertical axis based on the gravity vector determined from the accelerometer. If the foot is flat and still at time $t_{i}$ during one zero-velocity period, and after the step the foot is flat and still again at another time $t_{i+1}$, then the horizontal distance traveled between these two points is calculated as $d_{i}=\sqrt{{\left(x\left(t_{i+1}\right)-x\left(t_{i}\right)\right)}^{2}+{\left(y\left(t_{i+1}\right)-y\left(t_{i}\right)\right)}^{2}}$. The total horizontal distance traveled is then calculated as $d_{total}=\sum_{i=1}^{i=N-1}d_{i}$ where N is the number of periods when the foot was flat on the ground. Figure 2 illustrates the calculation of the total distance walked during a straight walk and turn while walking. The black dots show the position of one foot during the foot flat periods, the red trace shows the three-dimensional trajectory of the foot during each stride, and the black line segments show the forward, horizontal distance traveled during each of the three steps. The total walk distance calculation is performed separately for each foot. We report the final total walk distance as the average of the total walk distances estimated for each foot. The proposed algorithm only uses feet sensors to calculate the total distance walked. Another way to estimate the horizontal distance traveled is to calculate the velocity magnitude in the horizontal plane and then integrate it [45]. However, this will include the total curvature of the foot trajectories between steps, which is larger than the straight-line distance used in current clinical assessments. This is also longer than the horizontal movement of the center of mass of the body which does not follow a horizontal path with as much curvature as the feet.

### 2.4. Statistical Analysis

Study I. To investigate the percentage error in estimating the distance, we first subtracted the distance estimated by the proposed algorithm from a fixed distance of 400 m and then normalized it to 400 m to calculate the percentage error.

Study II. To investigate an error between a manually determined distance and a digital distance estimated by the proposed algorithm, we used an average absolute error rate (100 ∗ |Distance_manual_ − Distance_digital_|_average_/Distance_manual_average_). Here, Distance_manual_ is referring to the manually calculated total distance walked using a measurement tape and Distance_digital_ is referring to the objectively calculated total distance walked using the proposed algorithm. The agreement between the manually determined distance and the digital distance estimated by the proposed algorithm was also investigated using the Bland and Altman method [46], and the intraclass correlation coefficient (ICC), specifically ICC (2,1) [47]. All statistical analysis was performed using R Studio IDE Version 1.2.5019 software.

## 3. Results

**Figure 3:**
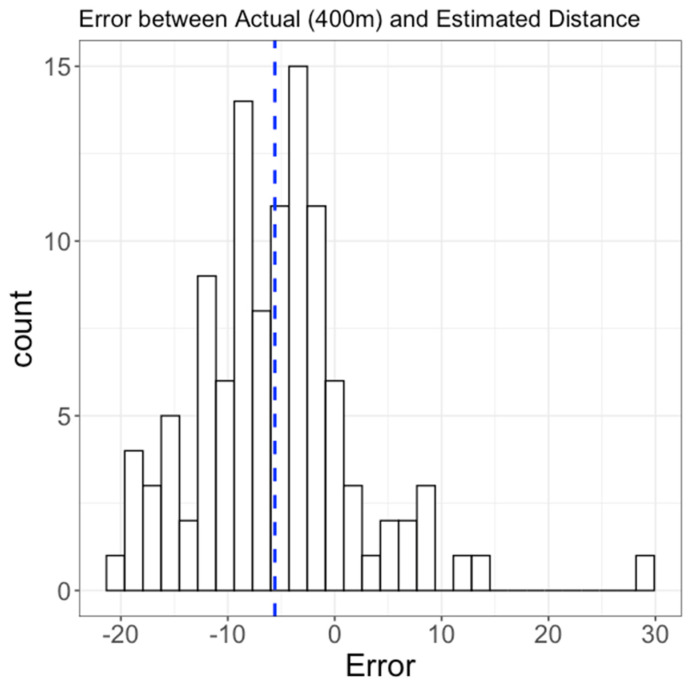
Histogram of error between 400 m and a total distance walked estimated by the proposed algorithm in 108 older adults (Study I). Vertical dashed line represents a mean of the histogram.

Study I. A total of 108 older adults (age = 71.20 ± 5.11 years; height = 176.86 ± 6.43 cm, weight = 93.32 ± 14.73 kg) participated in this study. The proposed algorithm showed an average absolute error rate of 1.92%, resulting in a slight underestimation from 400 m. The mean (SD) absolute error between the digital (algorithm) and 400 m walking protocol distance was 7.68 m (SD = 5.45 m; min = 0.18 m; max = 28.81 m). Figure 3 shows the histogram of the true error (distance_digital_—400 m) for all subjects.

**Figure 4:**
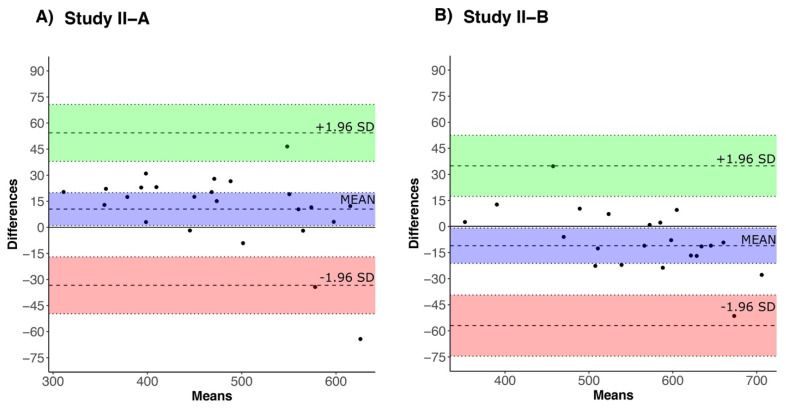
Bland-Altman plot for agreement between a manually calculated walk distance and walk distance estimates using the proposed algorithm for 6MWT in (**A**) Study II-A (15 m walkway distance) and (**B**) Study II-B (20 m walkway distance). Bias (mean), upper (+1.96 SD) and lower (−1.96 SD) limit agreement are represented by blue, green, and red colors, respectively.

Study II-A. A total of 24 subjects (20 PwMS and 4 HC) participated in this study. The proposed algorithm showed an average absolute error rate of 4.17% with respect to an average manual distance, resulting in an overestimation compared to the manual distance. The average absolute distance error between the digital and manual distance was 19.77 m (SD = 14.40 m; min = 1.80 m; max = 64.24 m) for 6MWT over an average manual distance of 474.42 ± 97.31 m. Further, the agreement between walk distance from the proposed algorithm versus manual distance was excellent (ICC_(2,1)_ [95% CI] = 0.97 [0.91–0.99]), with a bias of −10.50 [−19.94–−1.05] m, and upper and lower limits of agreement (LOA) of −54.34 [−70.71–−37.97], and 33.35 [16.98–49.73], respectively (Figure 4A).

Study II-B. A total of 22 subjects (9 PwMS and 13 HC) participated in this study. The proposed algorithm showed an average absolute error rate of 3.21% with respect to average manual distance resulting in a slight underestimation compared to the manual distance. The average absolute distance error between the digital (algorithm) and manual distance was 18.36 m (SD =18.79 m; min = 0.82 m; max = 83.85 m) for 6MWT over an average manual distance of 571.68 ± 103.24 m. Further, the agreement between walking distance from the proposed algorithm versus manual distance was excellent (ICC_(2,1)_ [95% CI] = 0.97 [0.91–0.99]), with a bias of 11.16 [0.52–21.80] m, and upper and lower limits of agreement (LOA) of −35.87 [−54.34–−17.41], and 58.19 [39.73–76.65], respectively (Figure 4B).

## 4. Discussion

In this study, we tested the validity and generalizability of a new distance-walked algorithm using wearable sensors on the feet in two different cohorts. Study I (*n* = 108) with a fixed distance walk test (400 m) in subjects over 65-years-old was used to develop the algorithm, while Study II with a fixed time walk test (6MWT) was used to validate the proposed algorithm in patients with multiple sclerosis. Study II had two sub-studies with different lengths of walkways, 15 m (Study II-A, *n* = 24) and 20 m (Study II-B, *n* = 22), respectively. The proposed algorithm achieved an absolute error rate of 1.92% for Study I, 4.17% for Study II-A, and 3.21% for Study II-B.

Our proposed algorithm does not require any information about the subject’s height, weight/age and does not need any calibration to calculate the total distance walked. In contrast, current commercial and custom algorithms rely on the availability of such information as a part of a calibration process [23,24,25,26,27,28,29,30,31,32,33,34,35]. Therefore, we believe this is a significant improvement. 

400 m walk test. Our results for the 400 m walk test are more accurate and consistent with the findings in the literature. Specifically, one study investigated the accuracy of the total distance walked with pedometers for the 400 m-walk test [30]. From all of the ten pedometers, the minimum error was observed for Sportline 345 (SL345) with a mean ± SD error (not absolute) of 12 ± 8 m with respect to 400 m fixed distance. Comparing the results of SL345, our proposed algorithm showed an improvement in the accuracy with a mean ± SD error of 6 ± 8 m. Both the pedometers and our proposed algorithm underestimated the total 400 m distance walked. Another study investigated the accuracy of the total distance walked during the 400 m-walk test with seven activity monitors [24]. Out of seven activity monitors, two overestimated the distance and the other five underestimated the 400 m distance. The authors reported a minimum error of 4.1 ± 8.1% for Fitbit Zip and Yamax CW-701 in contrast to our proposed algorithm that showed better accuracy (1.92%).

6-min walk test. Our results (average absolute error rate of 4.17% for Study II-A and 3.21% for Study II-B) are more accurate and consistent with the findings in the literature, albeit studies in the literature did not validate their algorithm in a separate cohort with a different protocol. Specifically, a recent study by Ata et al. [33] investigated the accuracy of the built-in iPhone distance-walked algorithm (with the iPhone placed in the hand) compared to the manually measured distance walked for the 6MWT in patients with peripheral artery disease. The authors found that the iPhone distance-walked algorithm overestimated distance with a bias of 43 ± 42%. In contrast, in the study by Juen et al. [35], the authors built a regression model from a smartphone and achieved an error of 5.87% for 6MWT. In a successive attempt, Juen et al. [34] further developed a machine learning model (using support vector machine algorithm) and achieved an error of 3.23% for 6MWT. To further improve the accuracy, Capela et al. [25] proposed an improved algorithm with a smartphone that achieved an average error of 0.12% for 6MWT. However, we recommend caution in interpreting the results as the authors used walkway length information to achieve this accuracy. Similarly, another study by Brooks et al. [28] developed a linear model of the distance-walked algorithm from smartphones and found an average error of 10%. Furthermore, when the same model was applied to two independent datasets, the authors found an average error of 10% (*n* = 33 in clinic) and 5% (*n* = 16 in home).

In Study II-B, we observed a single outlier where the calculated distance error was 83.85 m. This occurred in the subject who had the largest walk distance. Further investigation revealed that this was caused by the accelerations of the feet exceeding the bandwidth of the sensors, which was 48 Hz for the configured sample rate of 128 Hz. This was an unusual case because the subject was walking on a concrete floor, barefoot, at a rapid speed, with the sensors strapped firmly to the feet. The sensors were subjected to sharp acceleration impulses at the moments of foot strike and foot flat. This would not occur at the usual normal-paced walk, with more compliant flooring, or typical footwear designed to absorb this type of shock.

The average absolute error for Study II-A (15 m walkway) was 4.17%, and the average absolute error for Study II-B (20 m walkway) was 3.21%. On average, the algorithm slightly overestimated the distance in Study II-A and slightly underestimated the distance in Study II-B. We do not believe the bias was systematic, or that it was due to differences in the walkway distance. These are not large studies with hundreds of subjects, so it might just be due to the chance that one was overestimated and the other was underestimated. It might also relate to the length of the walkway and the effect of having some of the estimated distance include lateral distance during turns. However, the turns cannot be easily excluded because there are a variety of ways in which subjects approach a turn. For example, some subjects perform a pivot turn and rotate on the ball of their foot. Others will perform a broad turn in which they walk continuously in a semicircle without changing their pace. There is not an obvious, specific criterion that can be used to detect the beginning and completion of all turns to eliminate them from calculating the horizontal distance traveled from the sensors.

The proposed algorithm can be used even if the step detection algorithm produces false positives or false negatives. The algorithm calculates the distance traveled between stationary periods that correspond to the period when the foot is flat on the ground during the gait cycle. If a stationary period is not detected, the algorithm just calculates the distance traveled between stationary periods that are detected. This makes the algorithm insensitive to errors in step detection.

There are several limitations to the current study. First, our results were from a clinic test performed in the laboratory/controlled environment and should be repeated in an unsupervised environment. Second, we did not have the test-retest reliability of the total distance walked considering the participants’ own fluctuations during a day. Third, we had only a single clinical site’s data with three populations including patients with older adults with impaired fasting glucose, multiple sclerosis, and healthy control subjects. Future work is needed to validate the algorithm in a large multi-site clinical trial with test-retest reliability in different populations. Finally, the total distance traveled in turns may contribute to the errors in our studies, so one should be careful when comparing the results between studies with different walkway distances, which will necessarily change the number of turns included.

## 5. Conclusions

A novel algorithm was proposed to estimate the total distance walked using inertial sensors on feet. The walking distance algorithm was developed from 108 participants, and validated and tested for generalizability against different lengths of walkways in a total of 46 participants performing 6MWT. The results demonstrate the potential of an inertial sensor-based algorithm to estimate the total distance walked that can be used in a large clinical trial. Future work will validate the algorithm in remote settings with a large cohort of different populations.

## Data Availability

The data that support the findings of this study are available from the corresponding author upon reasonable request.
